# Comparison of ex vivo placenta perfusion and in vitro BeWo b30 cell models for assessing transfer of developmental and reproductive toxic compounds

**DOI:** 10.1007/s00204-026-04418-8

**Published:** 2026-04-28

**Authors:** Damian Roelofsen, Sandrine Spriggs, Beate Nicol, Petra van den Broek, Iris Muller, Hedwig van Hove, Frans Russel, Rick Greupink

**Affiliations:** 1 Department of Pharmacy, Pharmacology and Toxicology, Radboud University Medical Center, Nijmegen, 6525 GA The Netherlands; 2 Unilever Safety, Environmental and Regulatory Science (SERS), Colworth Science Park, Sharnbrook, Bedfordshire, MK44 1LQ UK

**Keywords:** Placental transfer, BeWo b30, Apparent permeability, Developmental and reproductive toxicology, Ex vivo Human placenta perfusion, Next generation risk assessment

## Abstract

There is an increasing interest in the prediction of exposure for risk assessment, as next generation risk assessment (NGRA) encompasses an exposure-led approach. Within the field of developmental and reproductive toxicology (DART), data on placental transfer is essential for estimating foetal exposure. However, human-relevant data is often limited or unavailable. Ex vivo human placenta perfusion is considered the gold standard in placental transfer research, but it is hampered by a low success rate, limited availability of tissue, and the inability to allow high throughput data collection. BeWo b30 cells can offer a high-throughput, lower-tier alternative to collect human-relevant placental transfer data. Here, thalidomide, valproic acid, amoxicillin, and antipyrine were tested in both model systems. A comparison matrix was derived to evaluate a set of shared placenta transfer parameter values obtained for each compound in the two systems. Transfer index (TI), relative transfer rate, initial transfer rate, and apparent permeability (Papp) were calculated for both experimental set-ups. The relative transfer rates in BeWo b30 cells and TIs of the placenta perfusions were highly comparable, with only a mean difference factor of 1.1. In addition, for the initial transfer rate and Papp values we found differences between the placenta perfusion and BeWo b30 experiments of an average factor of 4.5 and 2.4, respectively. Which parameter to calculate and extract from experimental data is determined by the underlying scientific question, e.g., whether a qualitative/relative or quantitative/absolute assessment of placental transfer is required. Nevertheless, the Papp values derived from both systems are particularly useful for parameterizing physiological-based kinetic (PBK) models and estimating foetal exposure following maternal external exposure, especially within NGRA.

**Supplementary Information:**

The online version contains supplementary material available at 10.1007/s00204-026-04418-8.

## Introduction

The placental barrier serves as a crucial interface between the maternal and foetal blood circulations. It plays an essential role in the exchange of nutrients, gases, and waste products (Burton & Fowden 2015). Next to the important role in regulating the transfer of endogenous substances, xenobiotics are also able to cross the placental barrier to different extents (Prouillac & Lecoeur 2010). The foetus is exposed to many different compounds, which can be of natural origin as well as chemicals that are present in the modern human environment, and therefore, chemicals should be tested for adverse effects on the developing foetus. To date, in vivo animal data remains the cornerstone of safety evaluation in regulatory toxicology, although they are increasingly questioned for their biological relevance for human effects (van der Zalm et al. 2022). However, an increasing number of alternatives are emerging within frameworks such as next generation risk assessment (NGRA), which aim to establish workflows that ensures robust risk assessment without the use of animal studies (Cote et al. 2016; Rajagopal et al. 2022; van der Zalm et al. 2022; Wood et al. 2024).

In the NGRA framework there is an increasing need for in vitro and ex vivo human-relevant new approach methodologies (NAMs), which is reflected by the exposure-led approach of NGRA to perform risk assessment (Wood et al. 2024). This paradigm shift, from an approach that primarily aims to assess hazard via the traditional high-dose in vivo animal studies, towards a more protective, exposure-driven approach, in which relevant exposure scenarios are taken into account, holds promise to improve context-based safety assessments (Rajagopal et al. 2022).

However, it is still challenging, if not impossible, to predict apical toxicological endpoints in a full human organism with a single NAM. This holds especially true for developmental and reproductive toxicological (DART) studies, given the vast complexity of the reproductive cycle with different sensitivity windows over time (Piersma et al. 2013). This has led to the development of so-called batteries of NAMs, which aim to capture DART processes (Schenk et al. 2010). These in vitro and in silico test batteries often include multiple toxicological endpoints and rely on in-vitro-in-vivo extrapolation (IVIVE) to link in vitro toxicological potency of compounds to relevant in vivo internal exposure. IVIVE can be done by using physiological-based kinetic (PBK) modelling approaches in order to estimate in vivo internal concentrations in plasma or tissues following external exposure (Piersma et al. 2013; van der Burg et al. 2015). Incorporating estimations of internal exposure into risk assessment opens the way for reverse dosimetry, which enables the translation of in vitro toxicological potency, i.e. points of departure (PoD), to calculate external exposures needed to reach such internal concentrations (Louisse et al. 2010). Additionally, IVIVE can be done by forward dosimetry, by calculating peak plasma concentration (C_max_) at a given dose, and comparing this to in vitro effect concentrations. Both the internal as well as external exposure can subsequently be used to calculate bioactivity:exposure ratios (BER), which can inform risk assessment (Kreutz et al. 2026; Mueller et al. 2025).

In the case of NGRA approaches to evaluate in utero developmental toxicity, it is necessary to link external exposure not only to estimations of maternal plasma concentrations, but also to expected foetal plasma and tissue concentrations. Data on placental passage of compounds is hence required to achieve proper estimates of internal exposure of the developing foetus. Placental transfer of chemicals can be estimated via different approaches. In silico models can take into account physicochemical properties of compounds and relate these to a general estimate of barrier permeability. Alternatively, in animal experiments, plasma concentrations at steady state can be compared to foetal or cord plasma concentrations. In terms of human-relevant models, the ex vivo human placental perfusion is considered to be the gold standard for the study of maternal-to-foetal transfer in the third trimester, because the 3D architecture of the placenta is temporarily preserved (De Sousa Mendes et al. 2016). For these experiments, a single unit of the placenta, called a cotyledon, is perfused from both the maternal and foetal sides to study transfer across the placenta. However, the system also faces limitations due to its labour-intensive design, low success rate, and dependency on fresh placental tissue. Given these limitations, the placental perfusion system alone is not sufficient in an NGRA framework, particularly due to the need for higher-throughput assays as a larger number of chemicals needs to be screened. Although multiple in vitro alternatives exist to study placental transfer (Dusza et al. 2023; Sastry 1999), one model considered suitable in this context is the in vitro BeWo b30 cell model.

The BeWo b30 cell line is a subclone of the original BeWo cell line, which is a human choriocarcinoma-derived trophoblast cell line (Liu et al. 1997). This subclone, in contrast to the original BeWo line, is able to form a confluent monolayer when cultured in an insert system under the appropriate conditions (Liu et al. 1997). The BeWo b30 insert system may therefore serve as a higher-throughput alternative to provide data on placental transfer of compounds compared to ex vivo human placenta perfusions.

The objective of this study was to compare the applicability of the BeWo b30 cell model, as an alternative to ex vivo human placenta perfusions, with 4 case study compounds, namely thalidomide, valproic acid, amoxicillin, and antipyrine. We used valproic acid and thalidomide, as both compounds are known to display good placental transfer and are known developmental toxicants. Where valproic acid is a straightforward compound in terms of stability and solubility, thalidomide is a challenging chemical to study as it shows spontaneous hydrolysis in aqueous buffers (Eriksson et al. 1992; Lepper et al. 2006). However, studying such a chemical allows to gain better insight into the handling of compound degradation in the model systems when estimating placental transfer. Amoxicillin was chosen as an example of a compound that is considered generally safe during pregnancy (Bookstaver et al. 2015). It is more hydrophilic than valproic acid and thalidomide and exhibits lower placental permeability. Antipyrine is a generally used reference compound for passive diffusion for which literature data has already been reported (Aengenheister et al. 2018; Dimopoulou et al. 2018; Li et al. 2013; Poulsen et al. 2009). We present a comparison matrix designed to support the interpretation of placental transfer data generated across these model systems for their application in risk assessment purposes. In this matrix, several ways to describe transport are included. The apparent permeability (Papp) is calculated because it is a widely used parameter to calculate transfer of compounds, particularly in in vitro cell systems. The transfer index (TI) values and relative transfer rates are used to compare the transfer of a compound relative to antipyrine (Li et al. 2013), while the foetal-maternal concentration (FM) ratio is often used to compare ex vivo placenta perfusion experiments between perfusions and across studies at the end of the experiments (Mose et al. 2008). Finally, the ratio for the initial transfer rate was calculated as comparison parameter for the initial linear part of the ex vivo and in vitro placental transfer experiments. In our in vitro study in BeWo b30 cells, the compounds were tested both in mixture and individually to study possible interactions.

## Materials and methods

### Reagents and chemicals

Dulbecco’s Modified Eagle Medium/F-12 Nutrient Mixture (DMEM/F12) with L-glutamine, without phenol red (21,041–025), 0.05% Trypsin–EDTA (25,300–054), and Penicillin (10,000 Units/mL) / streptomycin (10,000 ug/mL) (Pen Strep) (15,140–122) were purchased from Gibco (Eggenstein, Germany). Foetal bovine serum (FBS) (S-FBS-SA-015) was obtained from Serana (Pessin, Germany). The ZO-1 Monoclonal Antibody Alexa Fluor 488 (339,188) was supplied by Thermo scientific (Rockford, United States of America). Thalidomide (T144-1G) was obtained from Scientific Laboratory supplies (Fairham, United Kingdom). Potassium dihydrogen phosphate (A387573) was supplied by Merck (Darmstadt, Germany). Bovine Serum Albumin Fraction V (10,735,086,001) was obtained from Roche diagnostics GmbH (Mannheim, Germany). Acetic acid (6052) was supplied by Avantor Performance Materials (Deventer, The Netherlands). Sucrose (27,480.294) was obtained from VWR international (Leuven, Belgium). Dimethyl sulfoxide (DMSO) (D4540-100ML), 3-(4,5-Dimethyl-2-thiazolyl)-2,5-diphenyl-2H-tetrazolium Bromide (MTT) (M5655-1G), fluorescein sodium salt (NaF) (1,038,870,050), human collagen type IV (C5533-5MG), phosphate buffered saline (PBS), valproic acid (PHR1061-1G), amoxicillin trihydrate (31,586-250MG), antipyrine (A5882-25G), sodium chloride (S9625-1KG), potassium chloride (P9333-500G), magnesium sulfate heptahydrate (230,391-500G), sodium hydrogen carbonate (1.06329.1000), calcium chloride dihydrate (22,350–6), glucose (G7021-1KG), triton X-100 (23,472–9**)**, paraformaldehyde (P6148) and 4′,6-diamidino-2-phenylindole (DAPI, 32,670-5MG-F) were all obtained from Sigma Aldrich (Missouri, United States of America). Human serum albumin (Albuman) was obtained from Prothya Biosolutions, Amsterdam, The Netherlands. Heparin was purchased from LEO Pharma, Amsterdam, The Netherlands.

### Cell culture

The BeWo b30 cell line was kindly provided by Dr. Tina Burki (Empa, Switzerland) with permission from Dr. Alan Schwartz (Washington University, St. Louis, MO). The cells were cultured in DMEM/F12 medium supplemented with 10% FBS and 1% Penicillin/Streptomycin. BeWo b30 cells were maintained under a humidified atmosphere in the presence of 5% CO2, at 37 °C in T175 polystyrene cell culture flasks (Greiner Bio-One B.V., Alphen aan den Rijn, The Netherlands). Cells were routinely sub-cultured when confluency was > 80%, which was every 3–5 days. Cells were washed with PBS and detached using 0.05% trypsin–EDTA solution.

### Cell viability assay

BeWo b30 cells were seeded at a density of 1*10^4^ cells/well in a 96-well polystyrene plate (Cellstar, Greiner Bio-One B.V., Alphen aan den Rijn, The Netherlands) and placed in the incubator overnight to ensure cell attachment. The BeWo b30 cells were exposed to antipyrine, amoxicillin, valproic acid, and thalidomide using clinically-relevant C_max_ concentrations (1, 5, 50, and 1 µg/mL, respectively) for 24 h (Andrew et al. 2007; Jager-Roman et al. 1986; Teo et al. 2004; Zareba-Szczudlik et al. 2016). Crizotinib (100 µM), a known toxicant for BeWo cells, was used as positive control, cell culture medium served as negative control, and 1% DMSO was used as vehicle control (Eliesen et al. 2017). The final concentration in DMSO of all conditions was equal to or less than 1%. After 24 h, incubation solutions were removed and BeWo b30 cells were incubated with 0.5 mg/mL MTT dissolved in serum-free medium. MTT was removed after 2 h of incubation at 37°C and 100 µL of DMSO was subsequently added to dissolve the formed formazan crystals. Absorbance was measured at 570 nm and subtracted from the background signal of the sample determined at 690 nm and the absorbance of the blank determined at 570 nm using a Biorad Multiwell Plate reader (Veenendaal, The Netherlands).

### Transwell experiments

BeWo b30 cells were seeded at a density of 2*10^5^ cells / mL on polyethylene terephthalate (PET) inserts with 0.59 cm^2^ surface area (SA) and 0.4 µm pore size (BRAND GMBH Wertheim, Germany), placed in 24-well PET BRAND plates (BRAND GMBH Wertheim, Germany). Before cell seeding, the inserts were coated with 50 µg/mL human placental collagen type IV for 1 h at 37°C, after which they were washed with PBS. The apical side was filled with 0.4 mL and the basolateral side was filled with 1.5 mL of culture medium. The medium was replaced on both sides every 72 h. Transepithelial electrical resistance (TEER) measurements and NaF leakage assays were used to monitor the formation and integrity of the monolayer.

### TEER measurements

TEER measurements were performed to assess monolayer integrity over time. An Epithelial Volt-Ohm Meter (Millicell ERS-2) coupled with an adjustable electrode probe set (MERSSTX03, Millicell ERS-2) was used for all TEER measurements. The Epithelial Volt-Ohm Meter was regularly calibrated according to manufacturer’s instruction using the test electrode set (MERSSTX04, Millicell ERS). After seeding, daily TEER measurements were performed for 14 consecutive days to determine monolayer development of the BeWo b30 cells. These data were used to determine the optimal day after seeding to perform experiments. Next, for all experimental inserts, pre- and post-experimental TEER values were determined.

The plate with inserts was placed in an air flow hood for 30 min before starting the TEER measurements to let the medium adapt to room temperature. Concurrently, the electrode was sterilized in 70% ethanol at room temperature for 25 min before starting the TEER measurements. After sterilization, the electrode was air-dried and the system was checked before each series of measurement according to manufacturer’s instructions. The electrode was placed vertically in the inserts and resistance was read when the value given by the system was stable. For a detailed calculation on TEER values see the supplementary file.

### NaF leakage assay

To assess the permeability of the monolayer, NaF was added as marker to study the paracellular transfer. The transfer of NaF was assessed at day 3, 6, 9, and 12 post-seeding. In short, a 5 mM stock solution of NaF was diluted in DMEM/F12 medium (without phenol red) to a final concentration of 5 µM and 200 µL was added to the apical side of the insert. Next, 1000 µL of culture media was added to the basolateral compartment. Inserts were incubated for 3 h and three samples of 100 µL per insert were taken from the basolateral compartment. Samples were added to a black polystyrene 96-well plate (VWR international B.V., Amsterdam, The Netherlands) and concentrations were determined using the Victor X3 multiplate Reader (Perkin Elmer) with an excitation and emission wavelength of 485 and 535 nm, respectively. The percentage of compound measured in the basolateral compartment was calculated based on the start concentration of NaF added to the apical compartment.

### Compound transfer studies across the BeWo b30 cell layer

Experiments to study the transport of the compounds were performed at day 9 post seeding. Compounds were added in their final concentration, which was equal to the concentrations used in the viability assay, to the apical compartment. Transport was measured from the apical to the basolateral side. The compounds used in this study were added both as a mixture and separately at equal concentrations, to determine possible mixture effects. Samples of 50 µL were taken from the final compound solution at the start and from the apical compartment at the end (24 h) of the experiments. Sampling from the basolateral compartment was performed at multiple timepoints. All samples from the compounds in mixture and the thalidomide transport assays were buffered using a citric acid—sodium citrate buffer (pH 3.0) in a 10:1 sample to buffer ratio to reduce the pH of the samples below 6.0. The aim was to reduce the spontaneous hydrolysis of thalidomide (Eriksson et al. 1992). After a sample was taken from the basolateral compartment, the missing volume was replaced by an equal volume of fresh culture medium. For a detailed explanation of the Papp calculation see the supplementary file.

### Ex vivo placenta perfusion studies

Ex vivo dual-side placenta perfusions in a closed–closed configuration were performed to study the placental transfer of the compounds in a higher tiered approach. End-of-third trimester placentas were obtained from consenting women who underwent elective caesarean section or vaginal delivery. Approval was obtained from our institutional medical ethical committee (CMO Arnhem/Nijmegen File 2014–1397) and the study was confirmed not be subject to the Medical Research involving Human Subjects and in agreement with the Medical Treatment Contracts Act. The placenta perfusions were performed as described by Schakenraad et al. with slight modifications (Schakenraad et al. 2021). For a detailed overview of the method used see the supplementary file.

### Apparent permeability in placenta perfusions

The calculations of the Papp values for the placenta perfusion experiments were performed similar to the calculations of the BeWo b30 cells (can be found in the supplementary file), without the need to correct for a control as no artificial barrier is present in this experimental set-up. The total mass for each timepoint was calculated by starting with the total volume present in the circuit. For each timepoint, 3 mL (two times a 1.5 mL sample was taken at each timepoint) was subtracted from the volume as compared to the previous timepoint. The formula can now be written as:

$$\Delta Q_{n} = C_{n} * (V_{wn } - \mathop \sum \limits_{{}}^{n - 1} V_{s} ) + \mathop \sum \limits_{j = 1}^{n - 1} V_{s} * C_{j}$$

where ΔQn is the mass transported for each timepoint, corrected for the mass removed due to earlier sampling periods. Cn describes the concentration from the sample at time tn. The part of ${(V}_{wn }-{\sum }^{n-1}{V}_{s}$) described the volume of the compartment from which the sample was taken, with Vwn representing the volume of the compartment from which is samples and Vs describing the sampling volume (3 mL). The sum ${\sum }_{j=1}^{n-1}{V}_{s}* {C}_{j}$ is added to correct for the cumulative mass which is removed due to earlier samples taken in the period prior to tn (from t = t1 until t = tn-1). Vs described the sampling volume (3 mL) and Cj is the concentration of the samples taken prior to tn.

Next, the permeability (P) for each compound was determined by the same formula used previously for the BeWo b30 cells:

$$P = \frac{{{\raise0.7ex\hbox{${{\Delta Q}_{n} }$} \!\mathord{\left/ {\vphantom {{{\Delta Q}_{n} } {{\Delta t}}}}\right.\kern-0pt} \!\lower0.7ex\hbox{${{\Delta t}}$}}}}{{A* C_{0} }}$$

where ΔQn/ Δt is the rate of transport of the compound (ΔQn) over time (Δt) across the placental barrier in the perfused cotyledon, A is the SA of the perfused cotyledon, which is assumed to be 3371 dm^2^ based on the total SA of a placenta of gestational week 40 and assuming an average of 35 cotyledons in one placenta (Dallmann et al. 2017). Finally, C_0_ is the initial concentration present in the maternal (donor) side of the experiment.

### Compound stability and hydrolysis of thalidomide

As thalidomide undergoes spontaneous hydrolysis in aqueous solution, we aimed to capture and subsequently correct for this phenomenon in our transfer experiments, while testing the stability of the other compounds at the same time. Therefore, 3 replicates of the compound mixture were ran through the placenta perfusion system without a cotyledon in the system. Also, empty collagen-coated transwell inserts were incubated with DMEM/F12 media containing the mixture of compounds at 37°C. Finally, we performed stability tests using a glass flask with the compounds in mixture at 37°C to exclude non-specific binding to the plastics of the experimental systems. Samples were taken at equal timepoints as compared to the experimental set-ups. This data was used to correct the measured thalidomide concentration over time for degradation. A detailed explanation on the calculation can be found in the supplementary file.

#### Transfer index values and relative transfer rates:

TI expresses the extent of placental passage of the test compound relative to that of the reference marker antipyrine in ex vivo human placental perfusion experiments. The TI values were calculated for the ex vivo placenta perfusion, whereas the relative transfer rates were derived from the in vitro BeWo b30 data as was done previously (Li et al. 2013). For a detailed explanation on the calculations see the supplementary file.

#### Initial transfer rate and foetal-maternal concentration ratios

The initial transfer rate of the basolateral/foetal perfusate concentration and the FM ratio were determined as described in literature with slight modifications (Poulsen et al. 2009). For a detailed explanation on the calculations see the supplementary file.

#### Staining of tight junctions (ZO-1)

BeWo b30 cells were cultured for 9 days on inserts as described above. Cells were fixed in the inserts by adding 4% paraformaldehyde in PBS for 20 min at room temperature. Next, cells were permeabilized with 0.1% Triton X-100 in PBS for 15 min after which 3% BSA solution in PBS was added as blocking agent for 30 min. The anti-ZO-1 monoclonal antibody (ZO1-1A12, Invitrogen, 339,188) was dissolved in 1% BSA solution in a 1:50 dilution and added overnight to the inserts at 4°C. Finally, cells were washed with PBS and stained with 1 g/mL 4′,6-diamidino-2-phenylindole (DAPI) dissolved in PBS for 30 min at room temperature. Imaging was performed using a Zeiss LSM900 microscope and images were processed using Fiji (ImageJ 1.53t, NIH, USA).

#### Liquid chromatography mass spectrometry (LC–MS/MS)

All concentrations were determined using LC–MS/MS. For a detailed overview of the method see the supplementary file.

#### Statistical analysis

Data are expressed as arithmetic mean ± SD. Cell viability data was analysed using the Kruskal–Wallis Test combined with post-hoc testing using the Dunn correction for multiple testing. Multiple Mann–Whitney U testing with Bonferroni correction was used for the comparison of timepoints between in vitro transfer with compounds in mixture and when added separately. The Pearson’s correlation coefficient was determined for all initial transfer rates. Simple linear regression was performed on plotted data. All statistical analyses were carried out using GraphPad Prism version 10.4.1, and significance was set at p < 0.05.

## Results

### Barrier integrity

**Fig. 1:**
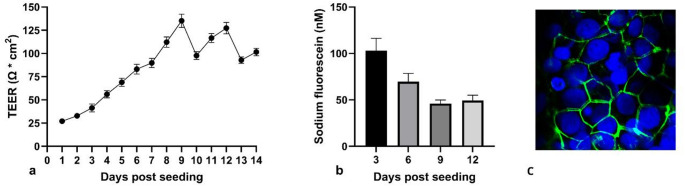
Overview of markers to study barrier development and integrity of the BeWo b30 cells. **a** shows the average TEER value for each day for 14 consecutive days after seeding. **b** shows the passage of NaF after 3 h at different days after seeding. **c** Confocal image of ZO-1 and DAPI staining for the BeWo b30 cells cultured in inserts for nine days, taken with a 63 × objective lens (63x/1.4 Oil Plan Apochromat), total magnification is 630x. green = ZO-1, blue = DAPI. Data is shown as mean ± SD, N = 3 biological replicates

Formation of the BeWo b30 cell layer was monitored over time using daily TEER and NaF measurements on days when media was replaced, since this minimized interference with the baseline culture conditions of the cells when grown in inserts. As shown in Fig. 1a, a steady increase of TEER values is observed from day 1 (27 ± 2 Ω*cm^2^) until 9 days after seeding (135 ± 7 Ω*cm^2^) the cells. From 9 days onwards there is a stable fluctuation in TEER values ranging between 93 to 127 Ω*cm^2^ observed. Figure 1b shows the NaF transfer over the developing cell layer at specific days after seeding. A decrease of NaF transfer is visible until day 9 post seeding, which stabilizes at day 12. Control experiments without cells showed no difference in NaF transfer in the inserts (see supplementary Figure S1). There was a high correlation between the increased TEER and decreased NaF concentration present in the basolateral compartment after three hours, with a R^2^ of 0.96 (see supplementary Figure S2). Finally, at day 9 post seeding well-defined tight junctions could be observed as can be seen in Fig. 1c. Therefore, for all cell experiments carried out after this evaluation, BeWo b30 cells were cultured in inserts for nine days before use.

### Compound toxicity and transfer

Amoxicillin, antipyrine and thalidomide did not affect cell viability at the tested concentrations. Valproic acid showed a small drop in cell viability at the clinically-relevant C_max_ concentration of 50 µg/mL as shown in Figure S3. However, as this concentration was at the lower end of the clinically observed plasma concentrations found in vivo and cell viability was still approximately 80%, this concentration was still used in the BeWo b30 transfer experiments as well as in placenta perfusion experiments.

Figure S4 and S5 (see supplementary file) shows the stability of the four tested compounds in both systems. It can be observed that antipyrine, amoxicillin and valproic acid are stable in both model systems. However, thalidomide disappears over time in both the placenta perfusion system as well as in the in vitro insert system. In Figure S4 (see supplementary file) there is no binding to the experimental systems as stability in glass showed equal results compared to the data from the experimental systems. This confirmed that the loss of thalidomide can be explained by spontaneous hydrolysis. Plotting Ln-transformation of the thalidomide data versus time yielded linear degradation kinetics with R^2^ of 0.93 and 0.99 for the placenta perfusion and insert system, respectively. This data was subsequently used to correct the transfer of thalidomide for the spontaneous hydrolysis.

**Fig. 2:**
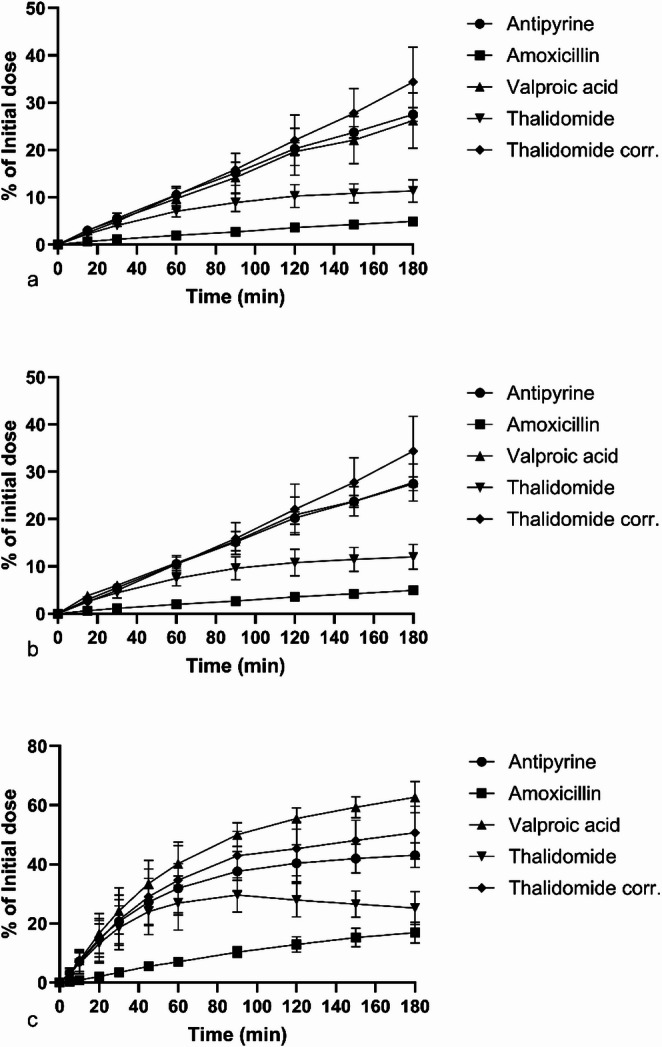
Overview of the percentage of initial dose present in the foetal/basolateral circulation at different timepoints. **a** Percentage of initial dose present in the foetal circulation at different timepoints in the ex vivo placenta perfusion system. Compounds were tested in a mixture. **b** Percentage of initial dose present in the basolateral compartment at different timepoints in the BeWo insert system. Compounds were tested separately. **c** Percentage of initial dose present in the basolateral compartment at different timepoints in the BeWo insert system. Compounds were tested in a mixture. Thalidomide corr. = thalidomide data corrected for spontaneous hydrolysis. Data is depicted as mean ± SD. N = 4 biological replicates for the ex vivo placenta perfusion and N = 3 biological replicates for the in vitro BeWo b30 assays

Figure 2 shows the percentage of the initial dose found in the basolateral/foetal compartment. From a qualitative ranking perspective, amoxicillin shows the lowest transfer in both systems, followed by thalidomide. In the placenta perfusion experiments (Fig. 2a), thalidomide corrected, which represents the thalidomide transfer data corrected for the spontaneous hydrolysis, showed slightly higher transfer as compared to antipyrine, followed by valproic acid demonstrating the highest transfer. Antipyrine and valproic acid show almost identical transfer in the BeWo b30 experiments after which thalidomide corrected presents slightly lower transfer (Fig. 2b and 2c). The transfer of the compounds was studied both as separate compounds as well as in one single mixture in the BeWo b30 in vitro assay to exclude possible interactions such as inhibition of transporters and/or metabolizing enzymes. None of the compounds showed a statistically significant difference between the compounds tested in mixture and tested separately, when a comparison was made at each timepoint of sampling. TEER values were measured pre- and post-experimentally. Figure S6 shows the overall pre- and post-experimental TEER values for all transfer experiments. Control experiments showed a pre- and post-experimental TEER of 135 ± 7 and 98 ± 4 Ω*cm^2^, respectively. For the compound in mixture, pre- and post-experimental TEER were found to be 128 ± 5 and 102 ± 6 Ω*cm^2^. When compounds were added separately, pre- and post-experimental TEER were found to be 130 ± 9 and 92 ± 7 Ω*cm^2^ for thalidomide, 128 ± 8 and 92 ± 7 Ω*cm^2^ for valproic acid, 128 ± 8 and 94 ± 14 Ω*cm^2^ for amoxicillin, and 124 ± 14 and 101 ± 8 Ω*cm^2^ for antipyrine.

Antipyrine and valproic acid reached equilibrium in the placenta perfusion experiments within 3 h and in the BeWo b30 system within 24 h, whereas amoxicillin did not reach equilibrium in either model systems within these experimental periods (see supplementary Figures S7-S9). The equilibrium for thalidomide could not be assessed on the original data. Based on the data corrected for hydrolysis, equilibrium would have been reached within 3 h for the placenta perfusion experiments. The 24 h sample in the BeWo b30 experiments could not be corrected due to the long sampling interval since the limited apical volume in the in vitro BeWo b30 experiments does not allow for more frequent sampling.

None of the compounds showed high accumulation in the placental tissue as can be seen from Figure S10 (see supplementary file). On average, all of the compounds showed less than 5% of the initial compound present in the placental tissue at the end of the experiment. Mass balance for the ex vivo placenta perfusion experiments was found to be 45 ± 6, 101 ± 8, 61 ± 17, and 80 ± 4% for thalidomide (uncorrected), valproic acid, amoxicillin and antipyrine, respectively. The mass balance found for the same compounds in the BeWo b30 was 4.0 ± 0.7, 102 ± 14, 72 ± 2, and 90 ± 3%, respectively. The fractions unbound as determined for the placenta perfusion experiments were found to be 75 ± 3, 25 ± 1, 90 ± 1, and 95 ± 2% for thalidomide, valproic acid, amoxicillin and antipyrine, respectively.

### Comparison of model systems

In Table 1 an overview of multiple analyses of the transfer data for the BeWo b30 and placenta perfusion experiments can be seen, including the fold difference between the specific parameter calculated for the placenta perfusion experiments as compared to the BeWo b30 assays. Papp was calculated at timepoint 20 min for the placenta perfusion and at 60 min for the BeWo b30 experiments as transfer was linear in time during these time intervals (slope in basolateral compartment > 0.95, data not shown). The largest difference between the placenta perfusion and BeWo b30 experiments can be seen in the initial transfer rate, where a mean difference a factor of 4.5 was found. On the other hand, a mean difference across all compounds of a factor of 1.1 was observed between the two experimental set-ups for the transfer indices and relative transfer rates. The Papp of the placenta perfusion experiments is calculated to be, on average, a factor 2.4 lower as compared to the Papp calculated for the BeWo b30 experiments for the compounds tested.

**Table 1:** Comparison matrix of different types of transfer parameters derived from the placenta perfusion and BeWo b30 data. For the BeWo b30 data, the top row represents the values as based on the compounds tested in a mixture. The bottom row represents the values as derived from the compounds tested separately. Papp = apparent permeability. FM = foetal-maternal. Data are given as mean ± SD. N = 4 biological replicates for the ex vivo placenta perfusion and N = 3 biological replicates for the in vitro BeWo b30 assays

	Antipyrine	Amoxicillin	Thalidomide (uncorrected)	Thalidomide (corrected)	Valproic acid
Papp calculation for placenta perfusion (cm/s), t = 20	7.9*10^–06^ ± 3.3*10^–06^	1.1*10^–06^ ± 0.4*10^–06^	7.1*10^–06^ ± 3.6*10^–06^	7.7*10^–06^ ± 3.9*10^–06^	8.9*10^–06^ ± 3.6*10^–06^
Papp calculated for BeWo b30 (cm/s), t = 60	2.3*10^–05^ ± 0.2*10^–05^	2.1*10^–06^ ± 0.01*10^–06^	1.5*10^–05^ ± 0.3*10^–05^	2.3*10^–05^ ± 0.4*10^–05^	1.8*10^–05^ ± 0.5*10^–05^
	2.4*10^–05^ ± 0.4*10^–05^	2.04*10^–06^ ± 0.01*10^–06^	1.6*10^–05^ ± 0.2*10^–05^	2.5*10^–05^ ± 0.2*10^–05^	1.2*10^–05^ ± 0.1*10^–05^
Fold difference (placenta perfusion / BeWo b30)	0.35	0.54	0.47	0.34	0.50
	0.34	0.55	0.45	0.31	0.75
Transfer index perfusion (t = 20)	–	0.17 ± 0.04	1.0 ± 0.1	1.0 ± 0.1	0.9 ± 0.1
Relative transfer rate BeWo b30 (t = 60)	–	0.18 ± 0.01	0.7 ± 0.1	1.0 ± 0.1	0.9 ± 0.2
	–	0.17 ± 0.01	0.74 ± 0.04	1.0 ± 0.1	0.8 ± 0.1
Fold difference (placenta perfusion / BeWo b30)	–	0.94	1.51	1.03	1.03
	–	0.94	1.36	1.04	1.22
Initial transfer rate (0–20 min for perfusion) (%/h)	45 ± 18	6.4 ± 2.1	40 ± 20	44 ± 22	50 ± 20
Initial transfer rate (0–60 min for BeWo b30) (%/h)	11 ± 0.6	2.0 ± 0.1	7.7 ± 1.7	10 ± 1.8	11 ± 1.3
	11 ± 0.5	1.90 ± 0.03	8.1 ± 0.7	11 ± 1.0	7.2 ± 0.6
Fold difference (placenta perfusion / BeWo b30)	4.21	3.14	5.15	4.20	4.55
	4.2	3.4	4.9	3.8	6.9
FM ratio perfusion (at 60 min)	0.6 ± 0.2	0.09 ± 0.02	0.6 ± 0.3	0.6 ± 0.3	0.6 ± 0.3

**Fig. 3:**
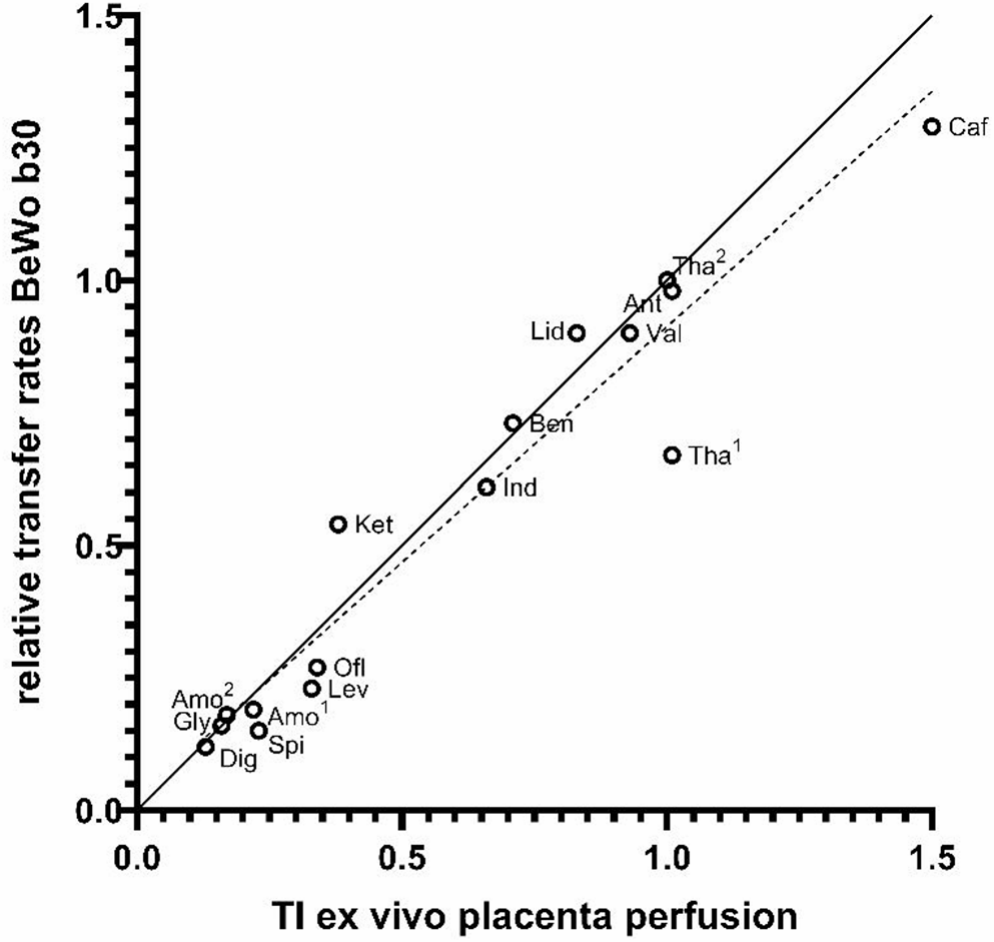
Plot of the TIs from the ex vivo placental perfusion model and relative transport rates from the in vitro BeWo model for the compounds retrieved from the paper of Li et al. 2013 combined with the compounds tested in this study (amoxicillin, valproic, acid and thalidomide). Amo^1^ amoxicillin data from this study, Amo^2^ amoxicillin data from Li et al 2013, Tha^1^ non-corrected thalidomide data from this study, Tha^2^ thalidomide data from this study corrected for spontaneous hydrolysis. Identity line is shown as solid line and the dashed line indicates the simple linear regression analysis

The plot (Fig. 3) shows the similarity between the TI values from the ex vivo placenta perfusions and the relative transfer rates from the in vitro BeWo b30 experiments. Overall, most datapoints are close to the line of identity (straight line) indicating similar outcomes between the ex vivo and in vitro data. Linear regression analysis (dotted line) showed a slope of 0.89 ± 0.07 and an intercept of 0.02 ± 0.05, indicating a slight underprediction of the BeWo b30 relative transfer rate compared to the ex vivo placenta perfusion TIs. The data taken from Li et al. 2013 with the addition of tested compounds from our experiments added, give a good overview of the correlation between the two model systems for thirteen individual compounds. Moreover, the compounds selected cover a broad range of chemical properties when considering logP (-3.0 to 4.5) and molecular weight (122.12 to 780.97), indicating that the BeWo b30 experiments might be a suitable lower tier approach or alternative when ex vivo placenta perfusion cannot be performed.

## Discussion

In this study, four compounds were studied in both the ex vivo dual side placenta perfusion and the in vitro BeWo b30 model systems. The monolayer formation of the BeWo b30 cells was monitored using daily TEER and NaF transfer measurements. A high linear correlation between the NaF transport rate and TEER values was observed, which is in line with findings of (Poulsen et al. 2009). These markers showed that the optimal day to perform transport experiments was 9 days after seeding, which was further strengthened by the clear presence of well-developed tight junctions as found by staining BeWo b30 cells grown in inserts 9 days after seeding for ZO-1.

Different articles report cut-off values on TEER values for the BeWo b30 monolayer to help decide when to commence transport experiments. Different cut-off values between 30 and > 160 Ω*cm^2^ have been suggested in literature (Ali et al. 2013; Mathiesen et al. 2014; Morck et al. 2010) (Albekairi et al. 2015) (Poulsen et al. 2009) (Li et al. 2013) (Kloet et al. 2015). Even higher pre-experimental TEER values between 70 and 394 Ω*cm^2^ have been reported (Dusza et al. 2022) (Aengenheister et al. 2018; Heaton et al. 2008; Liu et al. 1997), which are more in line with our own measurements. In our study, the test compounds had no effect on the post-experimental TEER, whether introduced in a mixture or individually, which indicates that the exposures did not seem to disrupt the monolayer integrity.

Several papers also report NaF transfer over the BeWo cells as a marker for paracellular transfer. Percentages reported to cross the cell layer are between 0.5 and 1.6%, partially depending on the different incubation times used (Dusza et al. 2022) (Aengenheister et al. 2018) (Poulsen et al. 2009) (Nielsen et al. 2011). Additionally, Papp values for passive diffusion control marker antipyrine are found to be between 2.6 and 11.2*10^–5^ (Aengenheister et al. 2018; Dimopoulou et al. 2018; Li et al. 2013; Poulsen et al. 2009). In this study, an average Papp of 2.3 ± 0.2*10^–5^ cm*s was observed, which is closely in line with some of the reported values. Overall, there is some variability in the different marker values reported, especially for TEER, to monitor BeWo b30 cell monolayer development. Differences in number of cells seeding, type of collagen, and type of inserts might explain part of the variability. However, our reported TEER and NaF values clearly showed an optimal day after seeding to perform transport experiments. Besides TEER and NaF transfer, the inclusion of antipyrine as an additional control marker allows to study inter-lab variability and might be used as reference/normalization marker in BeWo b30 transfer experiments. All these parameters highlight the importance of adequate model characterization and reporting of universally accepted reference markers to study inter-lab variability. This would be highly relevant to promote the acceptance of these new in vitro models for regulatory use in risk assessment.

Considering the compounds studied here, the spontaneous hydrolysis of thalidomide causes difficulties in interpretation of the data. The large difference in mass balance between the ex vivo and in vitro experimental set-ups can largely be explained by the difference in assay duration, since the placenta perfusion experiments were performed for 3 h, whereas the BeWo b30 experiment lasted for 24 h. However, it was uncertain whether the kinetics of this hydrolysis process are comparable between the ex vivo system and in vitro experimental conditions. Since differences in degradation kinetics can influence the correction rate for thalidomide, the kinetics of spontaneous hydrolysis were determined both in the in vitro as well as in the ex vivo system. The correction for hydrolysis of thalidomide data improved the correlation between the ex vivo and in vitro data for thalidomide transfer. Correcting for the hydrolysis increased placental transfer estimates. Determination of such kinetics for chemicals undergoing some form of degradation in the experimental system of interest can help in determining the impact it has on transfer and subsequently incorporate it in the calculation of placental transfer values. This is something that in vitro toxicologists should be aware of and ideally account for in both experiment setup and data analysis, as illustrated in the present study.

For valproic acid, Fowler et al. and Semczuk-Sikora et al. also performed ex vivo placental transfer experiments (Fowler et al. 1989; Semczuk-Sikora et al. 2010) and Fragki et al. performed transfer experiments in BeWo b30 cells (Fragki et al. 2022). Similar transfer kinetics can be observed when these studies are compared with the data retrieved here. Differences in time to equilibrium are likely caused by the difference in flow rate of the maternal circulation and the use of albumin in the experiments performed here. In vivo studies have reported that valproic acid concentration can be higher in foetal as compared to maternal circulation. This has been attributed to greater displacement from binding sites in the maternal blood due to increased levels of free fatty acids in the late stage of pregnancy (Fowler et al. 1989). The unbound fraction was significantly lower in cord serum as compared to maternal serum, with a strong correlation between free fatty acid concentrations and unbound valproic acid (Albani et al. 1984). Another possible explanation might be the involvement of a proton-linked monocarboxylate transporter (MCT) (Utoguchi & Audus 2000). Although a small but statistically significant decrease in cell viability was observed in the MTT assays at the clinically relevant concentration used in this study, no difference was found in post-experimental TEER as compared to control inserts without compound exposure. This indicates that the decreased cell viability observed in this study does not have a relevant impact on the in vitro transfer studies. Additionally, Ohyama et al. did not find a statistically significant effect on cell viability at a concentration of valproic acid of up to 1 mM (= 144.2 µg/mL) in BeWo cells (Ohyama et al. 2023), which indicates that the impact of the slightly reduced cell viability is likely limited.

Further elaborating on placental transfer values, different studies have retrieved different parameters from the type of experiments we conducted here (Li et al. 2013; Poulsen et al. 2009). Other studies have compared multiple in vitro placental cell lines, including co-cultures of BeWo b30 and HUVEC cells. However, in these studies, no comparison was made with ex vivo data and in the study of Aengenheister et al. it was concluded that horizontal shaking and co-culturing with HUVEC cells did not improve the standard BeWo b30 model for ranking by transfer (Aengenheister et al. 2018; Rothbauer et al. 2017). Building further on these studies, the extensive comparison done in this paper indicates that the transfer index exhibits the smallest difference between BeWo b30 cells and placenta perfusion experiments for the four compounds tested. The largest deviation was found for the values of the initial transfer rates of the basolateral medium and foetal perfusate concentrations. Although such parameters are useful to rank compounds in terms of extent of placental transfer relative to each other, they do not quantify placental transfer, which limits applicability in an NRGA context to estimate foetal exposure. Therefore, next to the commonly calculated Papp values for in vitro BeWo b30 experiments, we introduced a slightly modified set of equations to calculate Papp values for ex vivo placenta perfusion experiments. This value is deemed more practical for incorporation into a PBK model, allowing for foetal exposure estimates. This comparison directly enables evaluation of quantitative transfer values, which in turn can be used to directly assess the impact of the different experimental approaches on foetal exposure predictions. The most critical assumption we had to make to scale this new Papp value concerned the SA of the perfused cotyledon. The SA of perfused cotyledons is not easy to determine as one would need to perform a full microscopic analysis on each perfused cotyledon. As this was not within the scope of this study, we used a reported average SA value for a term placenta of 11.8 m^2^ based on multiple morphometric studies (Dallmann et al. 2017), which was divided by an average of 35 cotyledons in a placenta at term (Mian et al. 2021). The use of an average SA can lead to over- or underestimation of the actual SA present in the cotyledons used in the experiments as performed here. However, in risk assessment this can be taken into account by applying uncertainty analyses or worst-case scenarios for the placental SA assumption.

Interestingly, whereas the initial transfer rate shows faster transfer in the placenta perfusion, the Papp values show higher transfer in the BeWo b30 cells as compared to placenta perfusion experiments. This shows that the way experimental data is analysed partially influences the comparison between both model systems. The differences in initial transfer rate can likely be explained by the difference in experimental set-up, with the ex vivo placenta being continuously perfused, whereas the BeWo b30 cells remain in static (no flow) conditions (Poulsen et al 2009). The Papp values obtained under continuous flow in the placenta perfusion experiments do not lead to higher Papp values compared to the Papp values retrieved from the static BeWo b30 system. It is, however, likely that the difference in apparent permeability is partially explainable by the method of calculation and assumptions on placental SA as stated earlier. Next, when comparing the parameters calculated for the compounds added both in mixture and individually in the in vitro BeWo b30 assay, only minimal differences were observed. This indicate that the compounds can be added as a mixture without affecting the transfer rates derived from the assays. Using a mixture could therefore save significant time and resources when screening compounds for placental transfer. However, caution is warranted, since interactions on the level of transporters and/or metabolizing enzymes might be possible when investigating compounds in combination. Adequate literature review, supported by in silico QSAR screening, can help in determining the possible interaction risks (Cheng et al. 2011; Sedykh et al. 2013). Overall, when looking at the TIs and relative transfer rates for the ex vivo and in vitro model, a high correlation (P = 0.93) was observed when combining the data from this study with that of Li et al. 2013. The broad distribution in logP and molecular weight of the compounds studied, shows that the BeWo b30 model can produce very similar results in terms of TIs as compared to the gold standard ex vivo placenta perfusion.

In conclusion, we compared the in vitro BeWo b30 and ex vivo placenta perfusion experiments for a set of four compounds and calculated multiple parameters that provide an indication of placental transfer. As shown in this study, the comparison matrix enables a systematic evaluation of both model systems. The best agreement was observed for TIs and relative transfer rates, whereas the initial transfer rates described earlier differed the most. Nonetheless, all observed differences remained well below tenfold. Which parameter to calculate and extract from experimental data is inherently determined by the scientific question posed. Nevertheless, particularly in an NGRA framework, quantitative values, such as the Papp calculated for both systems here, are helpful for parameterizing PBK models and to estimate foetal exposure following maternal external exposure. Understanding the influence of experimental conditions and the data analysis strategy is essential for enhancing the applicability of in vitro assays in generating suitable quantitative placental transfer data.

## Supplementary Information

Below is the link to the electronic supplementary material.

Supplementary Material 1
