# Supplementary material for: Comparison of ex vivo placenta perfusion and in vitro BeWo b30 cell models for assessing transfer of developmental and reproductive toxic compounds
Source: Arch Toxicol. 2026 Apr 28;100(9):4185–97. doi: 10.1007/s00204-026-04418-8 (PMC13453992; doi:10.1007/s00204-026-04418-8)
Supplement: Supplementary file 1 [file 204_2026_4418_MOESM1_ESM.docx]

# Supplementary file

Comparison of ex vivo placenta perfusion and in vitro BeWo b30 cell models for assessing transfer of developmental and reproductive toxic compounds

## Journal

Archives of Toxicology

## Authors

Damian Roelofsen^1^, Sandrine Spriggs^2^, Beate Nicol^2✝^, Petra van den Broek^1^, Iris Muller^2^, Hedwig van Hove^1^, Frans Russel^1^, and Rick Greupink^1^

^✝^In memory of Beate Nicol, who passed away in July 2025

¹Department of Pharmacy, Pharmacology and Toxicology, Radboud University Medical Center, Nijmegen 6525 GA, The Netherlands.

²Unilever Safety, Environmental and Regulatory Science (SERS), Colworth Science Park, Sharnbrook, Bedfordshire MK44 1LQ, United Kingdom.

Corresponding author: Damian Roelofsen, [damian.roelofsen@radboudumc.nl](mailto:damian.roelofsen@radboudumc.nl)

***Ex vivo* placenta perfusion studies**

In short, an intact cotyledon was cannulated to re-establish both the foetal (6 mL/min) and maternal (12 mL/min) circulation within 45 minutes after arrival of the placenta at the lab. A 15-30 minute wash period was performed after cannulation, in which both circulations were washed in an open setting. After this washing period, both circulations were closed and the washing buffer was changed to the experimental buffer containing albumin. Next, the compounds were added to the maternal circulation in mixture. Experiments were considered successful when the maternal-to-foetal ratio for antipyrine at the end of the perfusion was >0.75. Samples were taken from the maternal and foetal buffer at multiple timepoints. All samples were buffered using citric acid - sodium citrate buffer (pH 3.0) in a 9:1 sample to buffer ratio to reduce the pH of the samples below 6.0 in order to reduce the spontaneous hydrolysis of thalidomide post sampling (Eriksson et al., 1992). The experiments were stopped after 180 minutes and the cotyledon was taken out of the system and weighed. Subsequently, three tissue samples were taken from different places of the cotyledon. Tissue samples were weighed and homogenized in perfusion medium containing 10% citric acid – sodium citrate buffer (pH 3.0) with a w:v ratio of 1:4 (tissue:perfusion medium containing buffer) directly at the end of the experiment. Homogenization was performed using a T10 basic Ultra-Turrax® disperser (IKA, Germany) for 20 seconds, three subsequent times while keeping the samples on ice. The homogenized tissue samples were stored at -20°C until further analyses.

**Calculation of transepithelial electrical resistance (TEER):**

The cell-specific resistance was calculated as follows:

$$R_{spec}= R_{cell}- R_{blank}$$

Where R_spec_ is the cell-specific resistance, R_cell_ is the resistance measured across a collagen-coated insert containing BeWo b30 cells and R_blank_ is the resistance measured across a collagen-coated insert without cells. All values obtained were in Ω. R_spec_ was subsequently used to calculate the TEER value corrected for SA of the insert:

$$TEER=R_{spec}* SA$$

Where TEER is the resistance value corrected for SA (Ω*cm^2^), R_spec_ is the cell-specific resistance and SA is the surface area of the insert (in cm^2^).

**Apparent permeability calculations for BeWo b30 experiments**

To calculate the apparent permeability the formulas below were used (based on Poulsen et al. 2009). First, the mass transported for each timepoint (${\Delta Q}_{n}$) into the basolateral compartment was calculated at each timepoint and was corrected for sampling volume due to previous sampling using the following equation:

$${\Delta Q}_{n}= C_{n}* V_{w}+ \sum_{j=1}^{n-1} V_{s}* C_{j}$$

Where ΔQn is the mass transported for each timepoint, corrected for the mass removed due to earlier sampling periods. Cn describes the concentration from the sample at timepoint tn and Vw is the volume of the compartment from which the sample was taken (either 200 µL or 1000 µL for the apical and basolateral compartment, respectively). The sum $\sum_{j=1}^{n-1} V_{s}* C_{j}$ is added to correct for the cumulative mass which is removed due to earlier samples taken in the period prior to tn (from t = t1 until t = tn-1). Vs described the sampling volume (50 uL) and Cj is the concentration of the samples taken prior to tn.

Next, the permeability (P) was calculated for each compound using the following formula:

$$P= \frac{\frac{{\Delta Q}_{n}}{\Delta t}}{A* C_{0}}$$

where ΔQn/ Δt is the rate of transport of the compound (ΔQn) over time (Δt) across the layer (calculated for inserts with and without BeWo b30 cells) at timepoint tn, A is the surface area of the membrane of the insert (0.59 cm2) and C_0_ is the initial concentration present in the donor side.

Finally, the permeability in the blank experiments and the experiments with BeWo b30 cells were used in the following equation to calculate the apparent permeability across the BeWo b30 cells alone:

$$P_{e}= \frac{1}{\left( \frac{1}{P_{c}}- \frac{1}{P_{b}} \right)}$$

where Pe is the apparent permeability across the BeWo b30 cells, Pb the permeability across the blank inserts (without cells) and Pc is the permeability across the inserts with BeWo b30 cells.

**Correction method for thalidomide degradation**

The measured concentrations of thalidomide were plotted on a natural logarithmic scale. Next, a linear regression analysis was performed on this data and a Pearsons correlation coefficient as well as an equation describing the linear regression was retrieved in the following form:

$$Ln (y) = Ax + B$$

Where Ln(y) represents Ln-transformed concentration of thalidomide and x the time of the experiment in minutes. This equation was used to calculate the total loss of thalidomide for each timepoint. Subsequently, the values were back transformed using Euler’s number as follows:

$$C (x) = e^{(y)}$$

Where C (x) is the concentration of thalidomide at timepoint x, e represents Euler’s number, and y is the Ln-transformed concentration of thalidomide at timepoint x. This value was used to calculate the percentage of compound left as compared to t=0 for each time point. This percentage was used to determine the concentration that would have been present at each time point if no hydrolysis had occurred.

**Calculations for the Transfer index values and relative transfer rates**

The relative transfer rate was calculated using data from the 60 minutes timepoint. The formula used to calculate this is as follows:

$$\frac{A_{f,x} \left( t \right) * A_{m,ap} (t=0)}{A_{f,ap} \left( t \right) *A_{m,x} (t=0)}$$

Where $A_{f,x} \left( t \right)$ and $A_{f,ap} \left( t \right)$ are the amount of compound X and the amount of antipyrine in the basolateral (foetal) compartment at timepoint t. Next, $A_{m,ap} (t=0)$ and $A_{m,x} (t=0)$ represent the amount of antipyrine and compound X on the apical (maternal) side at timepoint t=0.

Next for the *ex vivo* placenta perfusion, the TI values were calculated using a similar formula as compared to the relative transfer rate, with the only difference being that the maternal data is used at timepoint t and not t=0. This formula is as follow:

$$\frac{A_{f,x} \left( t \right) * A_{m,ap} (t)}{A_{f,ap} \left( t \right) *A_{m,x} (t)}$$

Where $A_{f,x} \left( t \right)$ and $A_{f,ap} \left( t \right)$ are the amount of compound X and the amount of antipyrine in the foetal circulation at timepoint t. Next, $A_{m,ap} (t=0)$ and $A_{m,x} (t=0)$ represent the amount of antipyrine and compound X present in the maternal circulation at timepoint t.

Finally, the TIs and relative transfer rates from the compounds present in the paper of Li et al. 2013 were extracted and the compounds tested in this manuscript were added to create a new correlation plot similar to the one in the paper of Li et al. 2013. Only the data from the *in vitro* studies without albumin present as described in the paper of Li et al. 2013 were used.

**Calculations of the initial slope and foetal-maternal compound ratios**

The initial slope for the *ex vivo* placenta perfusions was determined using the foetal perfusate concentration data from the first 20 minutes of the studies. For the BeWo b30 assays, data from the first 60 minutes of the basolateral concentrations were used. These intervals were chosen as permeation was linear for the time period (slope in foetal/basolateral compartment >0.95, data not shown).

The foetal-maternal (FM) compound ratio represents the ratio between concentration in the foetal circulation and maternal circulation at equal timepoints. These FM ratios were only calculated for the ex vivo placenta perfusion experiments as the experimental set-up of the BeWo b30 cells does not allow for apical sampling during the experiment. All FM ratios were calculated as follows:

$$FM ratio=\frac{C_{f}\left( t=60 \right)}{C_{m}(t=60)}$$

Where Cf and Cm are the concentrations in the foetal and maternal circulation of the ex vivo placental perfusion experiment, respectively.

**Liquid chromatography mass spectrometry**

Quantification of thalidomide, valproic acid, amoxicillin, and antipyrine in cell culture media, placental perfusion buffer, and placental tissue homogenates was performed using liquid chromatography-tandem mass spectrometry (LC-MS/MS). Free drug concentrations in perfusion buffer were determined using Rapid Equilibrium Dialysis (RED) single use plates (ThermoScientific, Rockford, USA) according to the manufacturer’s instructions. In short, placental perfusion buffer samples from multiple timepoints were loaded on one side of the RED and clean placenta perfusion buffer without albumin was added to the other side. This was incubated on a ThermoMixer C (Eppendorf Nederland B.V., Nijmegen, The Netherlands) at 250 rpm for 4 hours at 37 °C. The average from all measurements was taken to determine the average fraction unbound for each *ex vivo* placenta perfusion experiment.

For cell culture media, perfusate, homogenate and calibration samples, 50 µL of sample (or 50 µL standard) was mixed with 5 µL citric acid – sodium citrate buffer with a pH of 3, followed by 150 µL of ultrapure water. Internal standard solution (100 µL of IS in methanol) was added, containing 10 µg/mL thalidomide-d4, 5 µg/mL antipyrine-d3, 10 µg/mL amoxicillin-d4, and 100 µg/mL valproic acid-d6. Samples were vortexed thoroughly before adding 200 µL acetonitrile. After vortex mixing and centrifugation for 3 minutes at 21300 g, the supernatant was transferred to vials or plates for LC-MS/MS analysis.

Chromatographic and Mass Spectrometric Conditions Analysis was carried out using a Waters Acquity UPLC C18 BEH column (2z.1*50 mm, 1.7 µm particle size) maintained at 40°C. The mobile phase consisted of solvent A (0.2 mM ammonium fluoride in water) and solvent B (0.2 mM ammonium fluoride in a 75:25 methanol:acetonitrile mixture). A gradient program was used at a flow rate of 300 µL/min. Total run time was 6 minutes.

The tray temperature was maintained at 15°C. A 1 µL injection volume was used. Mass spectrometric detection was performed using a Waters Xevo TQ-S micro Triple Quadrupole Mass Spectrometer operating in both positive and negative electrospray ionization (ESI) mode, depending on the compound. MRM transitions and retention times used for the different compounds are given in table 1 and settings were as follow: Capillary voltage: +0.9 kV (ESI+), –1.0 kV (ESI–); Source temperature: 150°C; Desolvation temperature: 500°C; Desolvation gas flow: 1000 L/h; Cone gas flow: 50 L/h

Table 1: Overview of the MRM transitions and retention times for the different compounds.

| **Compound** | **Parent (m/z)** | **Product (m/z)** | **RT (min)** | **Polarity** | **Internal Standard** | **Parent (m/z)** | **Product (m/z)** |
| --- | --- | --- | --- | --- | --- | --- | --- |
| Thalidomide | 257.0 | 145.9 | 2.1 | ESI– | Thalidomide-d4 | 261.0 | 149.9 |
| Valproic acid | 143.0 | 143.0 | 3.1 | ESI– | Valproic acid-d6 | 149.0 | 149.0 |
| Amoxicillin | 366.1 | 113.9 | 1.2 | ESI+ | Amoxicillin-d4 | 371.2 | 114.8 |
| Antipyrine | 189.0 | 55.9 | 1.9 | ESI+ | Antipyrine-d3 | 192.0 | 59.0 |

Calibration curves were prepared by diluting DMSO stock solutions into Krebs buffer with 1% albumin (Krebs-Alb). Final calibration levels for thalidomide and antipyrine ranged from 0 to 1 µg/mL, for amoxicillin from 0 to 5 µg/mL, and for valproic acid from 0 to 50 µg/mL. Blank and quality control samples were analyzed alongside each batch to ensure adequate method performance.

**Fig. S1** Overview of the concentration of sodium-fluorescein in the basolateral compartment after 3 h for inserts without BeWo b30 cells. Data is depicted as mean+SD. N=3 biological replicates

**Fig. S2** Correlation between the TEER values and sodium-fluorescein concentration in the basolateral compartment at day 3, 6, 9 and 12 post seeding for the Beo b30 cells. Data is depicted as mean±SD. N=3 biological replicates

**Fig. S3** effect on cell viability of antipyrine, amoxicillin, valproic acid and thalidomide as measured with MTT after exposure for 24 h. Data is depicted as mean+SD. N=3 biological replicates

**Fig. S4** Overview of the stability of the tested compounds in the ex vivo placenta perfusion system and in a non-plastic set-up. a) stability of compounds in the ex vivo placenta perfusion system. b) stability in a glass Erlenmeyer set in a 37°C water bath. c) Ln-transformation of the stability data from the ex vivo perfusion system as well as from the stability tested in a glass Erlenmeyer at 37°C. Data is depicted as mean±SD. N=3 replicates

**Fig. S5** Overview of the stability of the tested compounds in the in vitro BeWo b30 system. a) stability of compounds in empty collagen-coated inserts used for the BeWo b30 transfer experiments. b) Ln-transformation of the stability data from the in vitro insert system. Data is depicted as mean±SD. N=3 replicates

**Fig. S6** Overview of pre- and post-experimental TEER values for all transfer experiments as performed with the BeWo b30 cells. a) data from day nine and ten post seeding for untreated BeWo b30 cells. b) pre- and post-experimental TEER data from the experiments where compounds were tested as mixture. c) pre- and post-experimental TEER data from experiments with antipyrine. d) pre- and post-experimental TEER data from experiments with amoxicillin. e) pre- and post-experimental TEER data from experiments with valproic acid. f) pre- and post-experimental TEER data from experiments with thalidomide. Data are depicted as mean+SD. N=3 biological replicates

**Fig. S7** Percentage of initial dose at different timepoints in the apical and basolateral compartment for the in vitro BeWo b30 transfer experiments over 24 h for compounds tested in mixture. a) transfer of antipyrine. b) transfer of amoxicillin. c) transfer of valproic acid. d) transfer of thalidomide. Data is given as mean±SD. N=3 biological replicates

**Fig. S8** Percentage of initial dose at different timepoints in the apical and basolateral compartment for the in vitro BeWo b30 transfer experiments over 24 h for compounds tested separately. a) transfer of antipyrine. b) transfer of amoxicillin. c) transfer of valproic acid. d) transfer of thalidomide. Data is given as mean±SD. N=3 biological replicates

**Fig. S9** Percentage of initial dose present in the foetal and maternal circulation at different timepoints for the ex vivo placenta perfusion transfer experiments over 3h for compounds tested in mixture. a) transfer of antipyrine. b) transfer of amoxicillin. c) transfer of valproic acid. d) transfer of thalidomide. Data is given as mean±SD. N=4 biological replicates

**Fig. S10** Overview of the percentage of the initial dose present in the perfused placental tissue after 180 minutes per successful experiment. Data is given as mean±SD. N=4 biological replicates.

**Fig. S11** Representative chromatogram of LC-MS/MS measurements for the 4 measured compounds with their internal standard.
